# Directional Locomotion of *C. elegans* in the Absence of External Stimuli

**DOI:** 10.1371/journal.pone.0078535

**Published:** 2013-11-05

**Authors:** Margherita Peliti, John S. Chuang, Shai Shaham

**Affiliations:** 1 Laboratory of Developmental Genetics, The Rockefeller University, New York, New York, United States of America; 2 Laboratory of Living Matter, and Center for Studies in Physics and Biology, The Rockefeller University, New York, New York, United States of America; Virginia Commonwealth University, United States of America

## Abstract

Many organisms respond to food deprivation by altering their pattern of movement, often in ways that appear to facilitate dispersal. While the behavior of the nematode *C. elegans* in the presence of attractants has been characterized, long-range movement in the absence of external stimuli has not been examined in this animal. Here we investigate the movement pattern of individual *C. elegans* over times of ∼1 hour after removal from food, using two custom imaging set-ups that allow us to track animals on large agar surfaces of 22 cm×22 cm. We find that a sizeable fraction of the observed trajectories display directed motion over tens of minutes. Remarkably, this directional persistence is achieved despite a local orientation memory that decays on the scale of about one minute. Furthermore, we find that such trajectories cannot be accounted for by simple random, isotropic models of animal locomotion. This directional behavior requires sensory neurons, but appears to be independent of known sensory signal-transduction pathways. Our results suggest that long-range directional behavior of *C. elegans* may not be driven by sensory cues.

## Introduction

Many organisms respond to starvation by altering their patterns of locomotion [1]–[4]. The general properties of some locomotory patterns, such as an area-restricted search performed immediately after removal from food, are conserved across species [5]–[7], suggesting that such patterns might reflect optimal behavioral strategies [8]. Theoretical studies have argued that locomotory patterns with straight relocation phases constitute a more efficient way of searching for food patches than simple diffusive motion [9]–[13], and, indeed, animal movement between patches has been observed to be more directional than movement within patches [14]–[16].

The free-living nematode *C. elegans* provides an excellent setting in which to investigate locomotory responses to external stimuli and to understand these in genetic and neural terms. On agar surfaces, *C. elegans* lies on its side, and moves forward by propagating dorso-ventral waves. Forward movement is occasionally interrupted by stereotypical behavioral sequences (‘reversals’ and ‘omega bends’) that result in sharp changes in the direction of motion [17]. In between sharp turns, the paths of *C. elegans* consist of gently curving segments, whose curvature is regulated by chemical and thermal stimuli [18]–[22]. Attractive stimuli also affect locomotory behavior by modulating the frequency of sharp turns, which is suppressed when the animal is climbing up a gradient, and *vice versa* [23]–[25]. At short times after removal from food, *C. elegans* has been shown to exhibit area-restricted search behavior [26]–[28].

While the characterization of the fine-grained components of *C. elegans* locomotion has received considerable attention, less is known about the geometrical and statistical features of its long-range trajectories. In particular, long-term locomotory behavior of *C. elegans* in the absence of stimuli has not been extensively examined. Previous studies have reported that, immediately after removal from food, *C. elegans’* motion has features of a random walk [21], [23] and that longer times of food deprivation induce suppression of turning behavior [26], [28]. However, experimental constraints in these studies limit the observation of animal trajectories to length scales of only a few cm.

Here, we provide a quantitative characterization of the trajectories of *C. elegans* in the absence of external stimuli over a period of about 1 hour. Our results suggest that animals can maintain directional movement over length scales of about 100 times their body length, and that they do so in spite of locally convoluted motion. We further demonstrate that such directionality requires sensory neurons, but not necessarily sensory transduction. Our results suggest the intriguing possibility that the long-range directional locomotion of *C. elegans* may not be driven by sensory cues.

## Results

### Imaging long-range movement patterns of *C. elegans*

**Figure 1:**
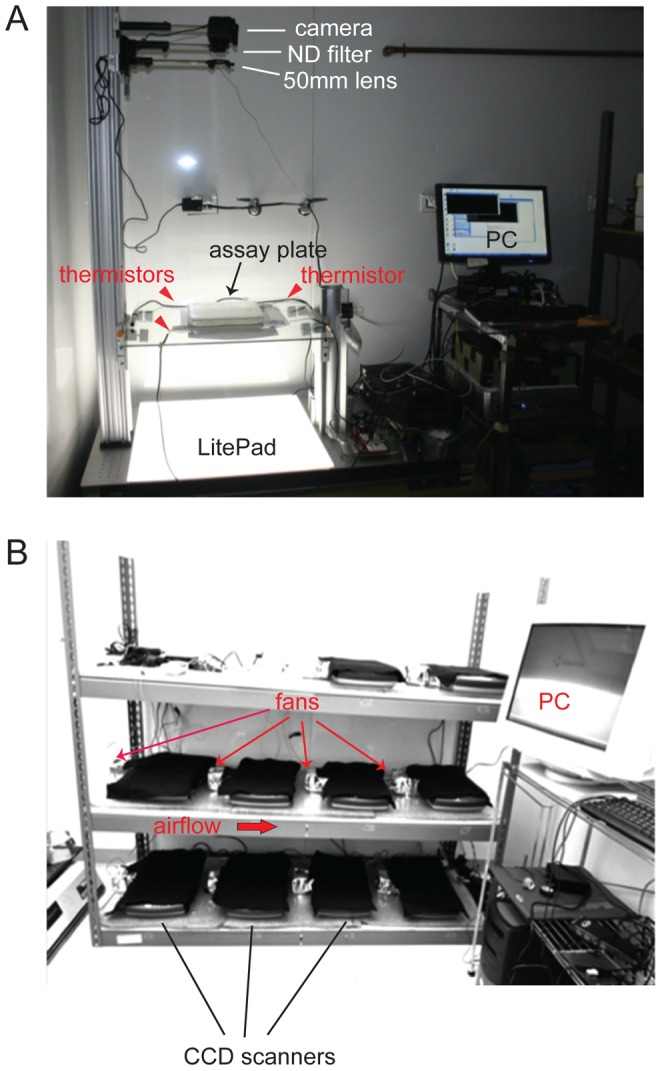
Imaging set-ups. (**A**) Camera imaging set-up. Light source: LED Lite-Pad (Rosco). ND: Neutral density filter. 50 mm lens: 50 mm focal length lens. Temperature was monitored with thermistors at the three corners of the plate indicated by arrowheads. (**B**) Scanner-array setup. 10 flatbed scanners are connected via USB connections to a PC running Linux. The red arrow indicates the direction of airflow within the scanner body.

To characterize the movement strategy of *C. elegans*, we tracked individual animals on large 22 cm×22 cm agar plates using two different imaging set-ups. One set-up, shown in Figure 1A, utilizes a consumer SLR camera placed at a 3 ft. working distance from the assay plate. This set-up provides relatively high temporal and spatial resolution (1 image per 1.5 s; ∼100 pixels/mm^2^), as well as homogeneous illumination and temperature conditions (see Materials and Methods).

The second set-up was developed to speed up data collection. It comprises an array of 10 flatbed scanners (Figure 1B), allowing for the parallel acquisition of 10 trajectories at a time. Image sequences on this set-up are acquired at lower spatial and temporal resolution than with the camera set-up (36 pixels/mm^2^; 1 image per 20 s). On both set-ups, we were able to track unrestricted animal movement for 30–80 minutes (see Materials and Methods).

### Directional motion, despite rapidly-decaying orientational memory

**Figure 2:**
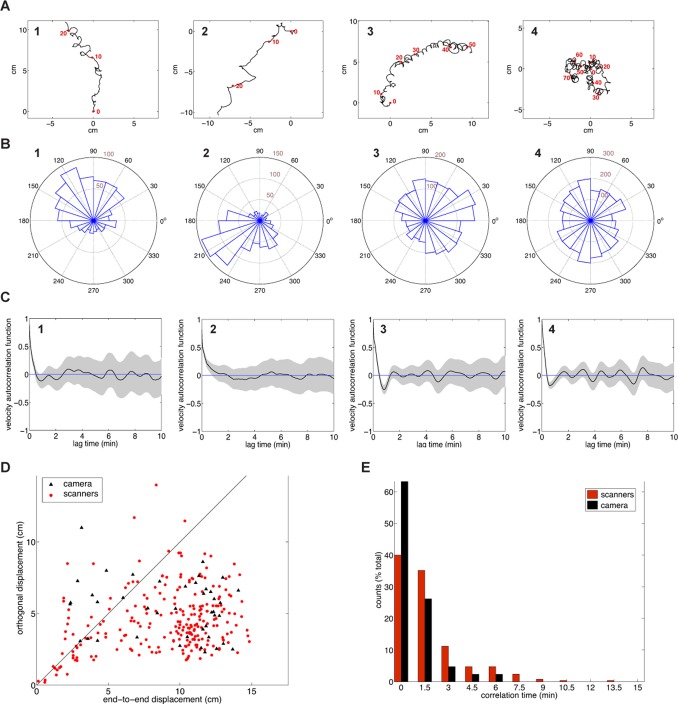
Trajectories of wild-type animals off food. (**A**) Trajectories of representative wild-type animals on the camera set-up. Red dots on the trajectory indicate the position of the animal at 10-minute intervals. (**B**) Histograms of ‘radial displacements’ for the trajectories shown in (**A**). Counts within a bin are obtained by summing frame-to-frame displacements corresponding to instantaneous headings whose direction falls within the interval specified by the bin. Directions are relative to the set-up. (**C**) Heading autocorrelation function. The function is defined, for a lag time *τ*, as 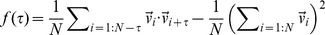, where 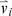 are the instantaneous unit direction vectors, and *N* is the total number of such vectors of the path. The function, smoothed with a cubic spline, is plotted in black; gray shading indicates the value of the signal +/- one standard deviation. (**D**) Scatter plot of the end-to-end displacement vs. the maximum displacement in the orthogonal direction for all trajectories. Red dots: scanner data. Black triangles: camera data. Black line: end-to-end displacement * = * orthogonal displacement. (**E**) Histogram of trajectory correlation times, defined as the first zero crossing of a path’s heading autocorrelation function. Red: scanner data-set; black: camera data-set. 75% of the scanner trajectories and 90% of the camera ones display correlation times of less than 3 minutes.

We collected 42 trajectories of individuals of the standard laboratory strain N2 with the camera set-up, and 250 with the scanner-array set-up. In both data sets, we noticed that a sizeable fraction of the paths maintained a directional course over time scales of several tens of minutes. Four representative trajectories obtained with the camera set-up are shown in Figure 2A. Trajectories 1, 2, and 3 display marked directional behavior, whereas no such effect is seen for trajectory 4. The directional bias of paths 1, 2, and 3 is reflected by a skew in the distribution of the distances traveled in a given directional range (Figure 2B), an effect not seen for trajectory 4. Paths collected with the scanner-array set-up display a similar distribution of features (Figure S1).

As a rough quantification of directionality, we initially examined the general shape of each track. Figure 2D shows, for every path in the data set, the displacement along the end-to-end vector, plotted against the total displacement in the orthogonal direction. Most trajectories appear skewed in the end-to-end direction.

Directional persistence may derive from long-term correlations in the instantaneous direction of motion. To test this hypothesis, and to identify the timescale over which *C. elegans* loses its orientational memory, we computed the autocorrelation function of the instantaneous headings. The results for the trajectories displayed in Figure 2A are shown in Figure 2C. The paths exhibit directional correlations over times of up to ∼1 minute. Surprisingly, however, no correlations could be detected over longer times. A similar pattern is displayed by the scanner paths (Figure S1). An analysis of the entire data set shows that 75% of the scanner trajectories, and 90% of the camera ones are characterized by correlation times of less than 3 minutes (Figure 2E). These observations demonstrate that long-range directionality occurs despite convoluted local movement.

### Directionality is independent of environmental and plate parameters

**Figure 3:**
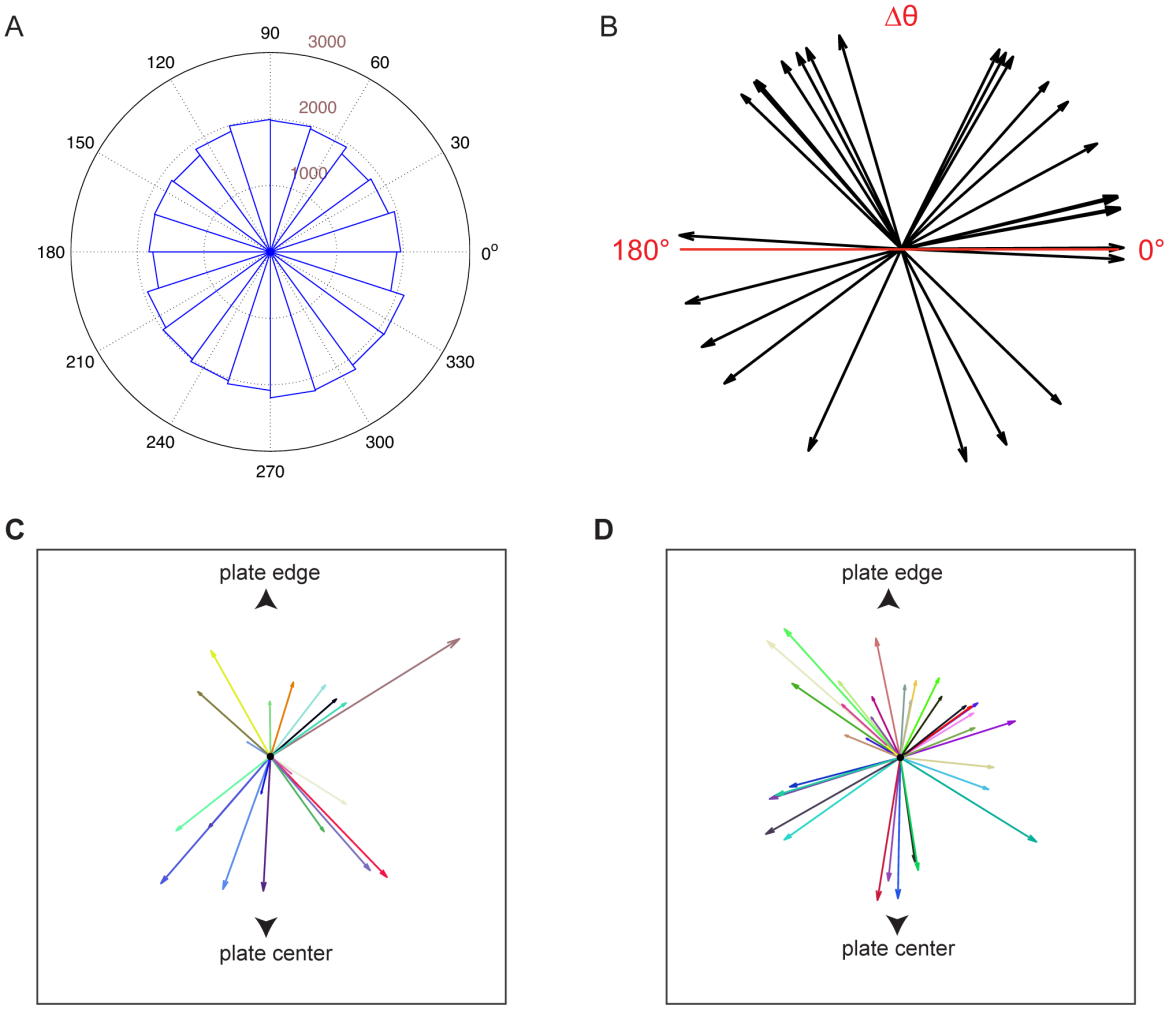
Directionality is independent of plate parameters. (**A**) Histogram of radial displacements (see Figure 2) for the entire data set (N  =  250 trajectories) acquired with the scanner-array set-up. (**B**) Relative direction Δθ of the paths of two animals assayed on the same plate in close succession. The direction of a path is defined as the direction of the end-to-end vector for the path. 0° corresponds to two paths that are parallel to each other, 180° to anti-parallel paths. N = 26 pairs. (**C**, **D**) End-to-end vectors of paths of animals started from intermediate positions between the center and the edge of a plate. (**C**): Freshly poured plates. (**D**): 12-day-old plates.

One explanation for directional persistence is migration towards an external signal. If this were the case, the distribution of path directions should reveal a bias towards a specific sector of the set-up. However, we did not detect such a bias (Figure 3A). Directionality could also arise in response to plate-specific cues. To test this, we examined the movement of individual animals placed sequentially on the same plate. As shown in Figure 3B, path orientations displayed no detectable correlation.

**Figure 4:**
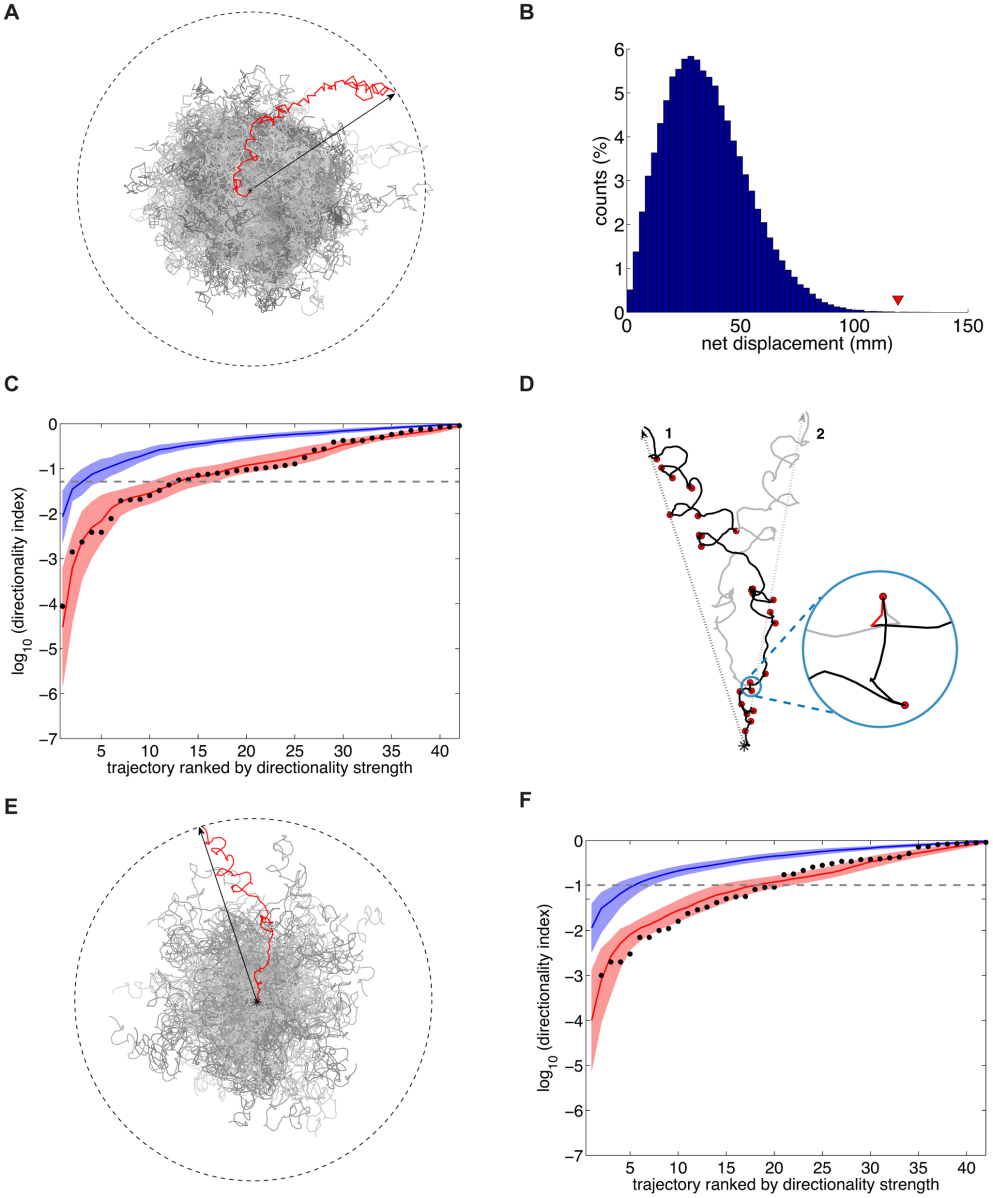
Random, isotropic motion does not account for directionality. (**A**) Synthetic paths for the correlated random walk (‘CRW’) model were obtained by sub-sampling an experimental path at its correlation time, and by drawing at random from the step sizes and turning angles distributions of the resulting path (experimental path, red; synthetic paths, gray). Black arrow: experimental net displacement. (**B**) Histogram of net displacements for 100,000 synthetic paths corresponding to the trajectory in (**A**). The red triangle indicates the experimental value. The directionality index of a path equals the probability of obtaining, from the model, a larger or equal net displacement than the experimental one. (**C**) Directionality indices for the camera and the scanners data sets. Indices are plotted by rank (from most to least directional). Black dots: camera data. Red line: average of 20 samples from the scanner-array data set (‘average scanner data set’). Red shading: average scanners data set +/− one standard deviation (‘std’). Blue line: average of 20 samples from the CRW model. Blue shading: average of the CRW model +/− one std. (**D**) ‘Mirror’ model. The experimental path (in black) is segmented into ‘runs’ and ‘turns’ (red dots). Synthetic trajectories are obtained by inverting turns with probability ½, and by reassembling runs and turns by continuity (see Materials and Methods). One such path, obtained by inverting the turn highlighted in circular inset (red line), is displayed in gray. Black asterisk: position at time 0. (**E**) The directionality index of a path is defined as in (**B**). (**F**) Directionality indices for the mirror model. Indices are plotted by rank. Black dots: camera data. Red line: average scanners data set (CRW model). Red shading: average scanners data set +/− one std. Blue line: average of 20 mirror model samples. Blue shading: average mirror model +/− one std.

We next asked whether a radial cue within the plates might account for directed motion. We placed animals at an intermediate position between the center and the edge of the plate. If a radial gradient exists, animals should move preferentially towards or away from the closest edge of the plate. However, as shown in Figure 3C, no such preference was detected on either freshly-poured or 12-day-old plates, in which a radial evaporation gradient might be expected. Moreover, paths obtained in these assays display similar quantitative features to paths obtained in the experiments described in Figure 2 (Figure 4, Figure S2).

### Random, isotropic motion does not account for directionality

Could the observed long-term directionality arise from random, isotropic movement? To address this question, we generated a set of 100,000 synthetic trajectories for every experimental path in the data set. Synthetic trajectories were constructed by first sampling the original path at its correlation time (see Materials and Methods), and then by drawing at random from the distributions of step sizes and turning angles of the sampled path, for an equal number of steps (Figure 4A). This model is in effect a correlated random walk, since the distribution of turning angles is usually not uniform [29]. We then compute the distribution of net displacements displayed by the set of synthetic trajectories, and compare it to that of the corresponding experimental path (Figure 4B). From such distribution we define the ‘directionality index’ of an experimental path as the probability of obtaining, from a single synthetic run, a larger or equal net displacement than the experimental one.

Figure 4C shows the directionality indices displayed by the camera and scanner-array data sets. The indices are shown together with those that would result from a set of synthetic paths obtained from the correlated random walk (‘CRW’) model. Directionality index values were plotted by rank, *i.e.* ranging from the smallest (most directional) to the largest (least directional). 29% of the scanner paths and 28% of the camera paths display indices smaller than 0.05, versus the 5% that would be expected, by definition, from an ensemble of CRW paths. Both data sets are significantly divergent from random (*p* = 10^−21^ for the scanner data-set, and *p* = 2⋅10^−4^ for the camera data set, respectively; Kolmogorov-Smirnov test).

By construction, our synthetic trajectories do not preserve the temporal structure of turning events of the original path, and destroy potential correlations between local displacement and heading. We thus decided to compare the camera data set to a more conservative ‘mirror’ model of random motion, one preserving to a greater extent the local features of our experimental paths. First, experimental tracks are segmented into bouts of smooth motion (‘runs’) and episodes of sharp turning (‘turns’) (see Materials and Methods). Then, for every turning episode, we invert, with probability ½, the portion of the path corresponding to the turn. Finally, we re-assemble the different segments of the synthetic path (‘runs’ and ‘turns’) in such a way that the directions at the ends of the segments coincide (Figure 4D). This procedure effectively amounts to building local mirror images of the track, resulting in sets of synthetic paths (Figure 4E) with the same local features as the experimental trajectories.

To quantify the experimental divergence from such ‘mirror’ model, we computed, as before, the probability of obtaining a larger or equal net displacement from a set of synthetic paths. The results, shown in Figure 4F, are in agreement with those obtained for the CRW model: 33% of the camera paths display probabilities smaller than a 0.05 threshold, and the data shows a significant divergence from the mirror model (*p* = 0.006, Kolmogorov-Smirnov test). Importantly, these results demonstrate that changes in turning frequency along the trajectory path cannot account for the directionality we observe, since the experimental turning frequency distributions are preserved in the mirror model synthetic paths.

### Emergence of directional behavior at long timescales

**Figure 5:**
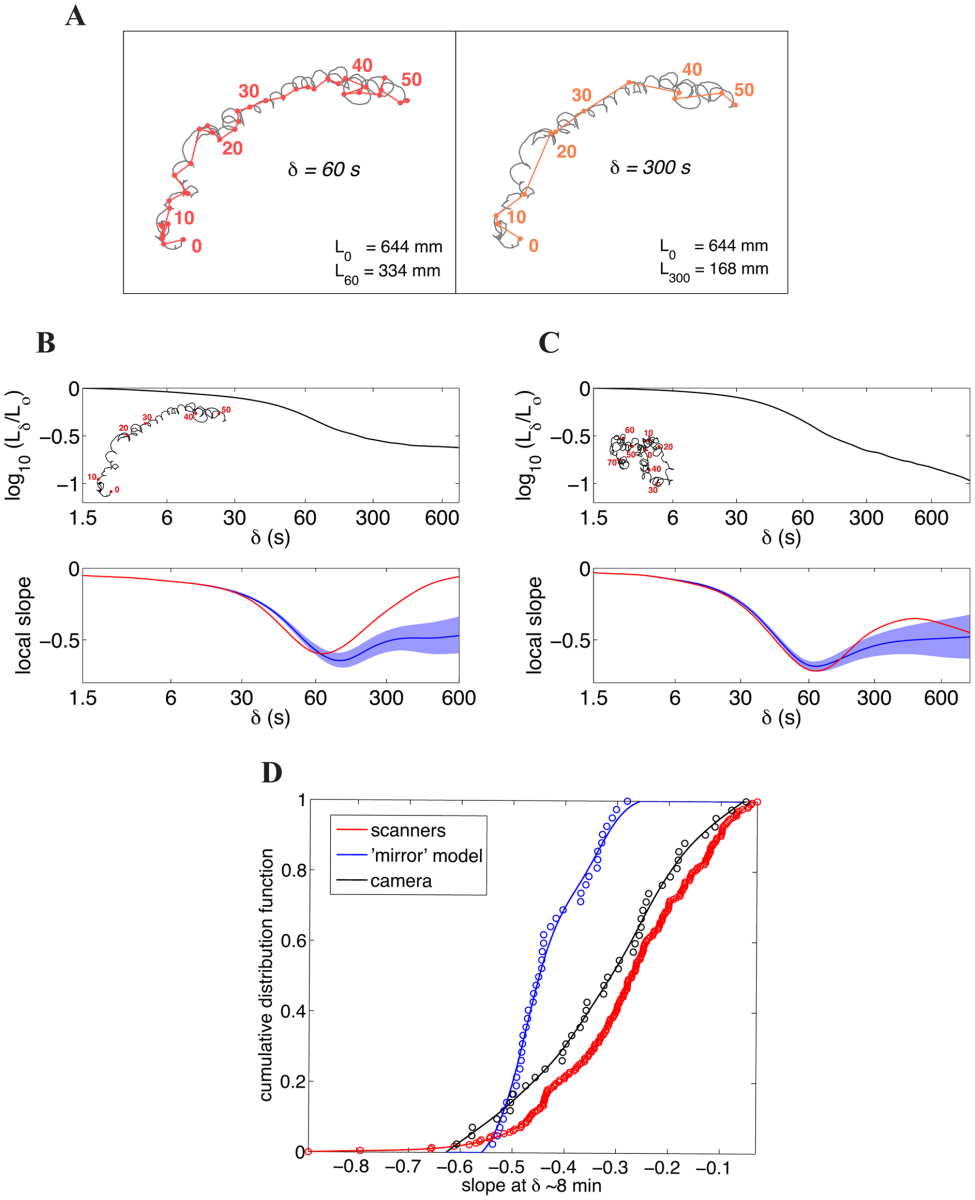
Emergence of directional motion at long timescales. (**A**) Coarse-graining procedure. Left: experimental path (black) coarse-grained at 60s intervals (red segments). Right: experimental path (black) coarse-grained at 300s intervals (orange segments). (**B**) Scaling behavior of a directional trajectory. Top panel: Scaling of the lengths of coarse-grained paths, in log-log scale. Paths are sub-sampled (‘coarse-grained’) at increasing time intervals *δ,* and their resulting length *L_δ_* is plotted against *δ*. Lengths are normalized to the total length
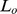
of the original (*i.e.* unsampled) path. Bottom panel: local slope of the plot of the logarithm of the coarse-grained length vs. the sampling interval *δ*. Red line: slope of the black line in the top panel. Blue line: average scaling of a set of mirror trajectories. Blue shading: average of the mirror model +/− one standard deviation. (**C**) As in (**B**), for a non-directional trajectory. (**D**) Cumulative distribution of the scaling exponent of the coarse-grained lengths at long times (*δ* ∼ 8 min). Black circles: camera data. Black line: cubic spline interpolating the camera data. Red circles: scanner data. Red line: cubic spline interpolating the scanner data. Blue circles: set of mirror trajectories. Blue line: cubic spline interpolating the mirror data. *p* value against the mirror model  =  10^−11^ (scanners data-set), *p* = 10^−4^ (camera data-set), Kolmogorov-Smirnov test.

To identify the time scale over which an animal’s behavior crosses over to directional motion, we took an approach borrowed from fractal analysis. For every experimental path, we construct a set of trajectories (‘coarse-grained’ trajectories) by sampling the original path at intervals of size *δ* (Figure 5A). We then compute the lengths
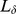
of these coarse-grained paths by summing the length of segments joining the points that comprise the path, and normalize these sums by the length 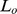 of the original path. This procedure is analogous to measuring the length of a coastline using a ruler of size *δ*. While a coastline’s length is not a uniquely defined quantity, as it depends on the size of the ruler utilized to measure it, the functional relation between the length 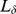
– measured at a scale *δ* – and *δ* itself is invariant. This relation can be expressed as 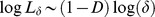, where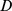 is the curve’s fractal dimension, a quantity that defines the degree of convolution, or space-filling, of the curve [30].

In the top panels of Figure 5B and Figure 5C we plot the behavior of 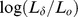 versus 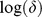 for two distinct trajectories (shown in the insets). At short times (*δ*<30 s) both paths exhibit locally directed motion, as reflected by the nearly horizontal trend of the curves. Sampling the data at longer times (*δ* ∼ 60 s), when correlations in directions have mostly decayed to zero, results, for both trajectories, in coarse-grained paths that are now more random. Accordingly, the logarithm of the lengths shows, in both cases, a drop towards larger negative values. At longer times (*δ* > 300 s), however, the behaviors of the two paths diverge. The curve corresponding to path 1 flattens out, revealing directionality. By contrast, no such effect is seen for path 2. Therefore, directed motion corresponds to local values of the slope close to zero (bottom panels of Figure 5B and Figure 5C). Corresponding sets of mirror synthetic trajectories display, instead, slopes in the neighborhood of −0.5, as expected for a diffusive process (bottom panels of Figure 5B and Figure 5C).

To examine the behavior of our entire data set using this analysis, we plotted the cumulative distribution of slope values at long times (Figure 5D). The slopes for the camera (black) and scanner data-sets (red) are displayed together with those for a set of mirror synthetic trajectories (blue). A large fraction of the wild-type data set displays directional behavior at long times; indeed, 50% of the wild-type distributions exhibit slope values greater than -0.3, compared to fewer than 1% of the synthetic trajectories. Consistent with our previous analyses, the scanner and camera data sets are statistically indistinguishable in their long-term behavior, and significantly more directional than the mirror model (*p* = 10^−11^ and *p* = 10^−4^ respectively, Kolmogorov-Smirnov test).

### Behavior of sensory mutants

To begin to understand the neural basis for directionality, we investigated the behavior of animals with defects in the nervous system, addressing specifically the role of sensory neurons, which regulate locomotory output even in spatially and temporally homogeneous environments [26]–[28]. Most sensory neurons in *C. elegans* have non-motile sensory cilia; accordingly, genetic lesions that affect cilia formation impair most sensory behaviors, with the exception of thermotaxis [31]–[33].

In Table 1 we summarize the results obtained for two cilia mutants, *che-2* and *daf-19*. Animals mutant for *che-2* have been reported to display confined tracks off food, resembling those of wild-type animals on bacterial lawns [26], [34]. Consistent with this observation, *che-2* animals fail to display directional behavior (*p* against the wild type  =  0.003, Kolmogorov-Smirnov test; Figure S3A). By contrast, *daf-19* mutants exhibit only a partial directionality defect, with a subset of the tracks displaying wild-type directional behavior (*p* against the wild type  =  0.03, Kolmogorov-Smirnov test; Figure S3B). This observation is surprising, given that mutations in *daf-19*, a transcriptional activator of *che-2,* result in the complete absence of sensory cilia, a defect more severe than that exhibited by *che-2* animals [33], [35]. Indeed, we confirmed the complete absence of cilia in two *daf-19* animals displaying directed motion by performing serial-section electron microscopy on such animals. Taken together, these results suggest that, while sensory neurons may be important for long-range directional behavior, sensory transduction is not essential.

**Table 1:** Behavior of sensory and pheromone mutants.

Genotype	Gene function	Sensory defect	Directionality	p-value
*che-2*	cilia formation	chemotaxis (‘ctx’)	defective	0.003 (vs. WT)
daf-19*	cilia formation (master regulator)	chemotaxis (‘ctx’)	partially defective	0.03 (vs. WT)
*osm-9*	sensory transduction (TRPV-related channel)	avoidance of repulsive compounds; nociception; ctx to some volatile compounds	wild-type	0.0008 (vs. CRW)
*tax-4(p678)*	sensory transduction (cGMP-gated channel)	thermotaxis; aerotaxis; ctx to some volatile and soluble compounds.	defective	0.008 (vs. WT)
*tax-4(ks28)*	sensory transduction (cGMP-gated channel)	same as *tax-4(p678)*	defective	0.006 (vs. WT)
*tax-2*	sensory transduction (cGMP-gated channel)	same as *tax-4(p678)*	defective	0.009 (vs. WT)
*gcy-8;gcy-18;gcy-23*	sensory transduction (guanylyl cyclases)	thermotaxis	wild-type	0.0003 (vs. CRW)
*gcy-31;gcy-33;gcy-35*	sensory transduction (guanylyl cyclases)	aerotaxis	wild-type	0.003 (vs. CRW)
*gcy-36::*EGL-1	sensory neurons ablated	aerotaxis	wild-type	0.0002 (vs. CRW)
*odr-1*	sensory transduction (guanylyl cyclase)	ctx to volatile compounds sensed by TAX-4 neurons; phototactic avoidance	wild-type	0.004 (vs. CRW)
*che-1*	sensory transduction (specification of sensory neuronal fate)	ctx to soluble compounds	wild-type	0.02 (vs. CRW)
*daf-22*	*C. elegans* pheromone synthesis	*dauer* formation	wild-type	0.01 (vs. CRW)

Significance is estimated with respect to the wild-type scanner-array data-set (‘WT’) or to the correlated random walk model (‘CRW’). *daf-19 mutants were assayed in a *daf-16* background to bypass constitutive *dauer* formation.

To further explore this issue, we examined the effect of genetic lesions impairing the activity of sensory neurons. We assayed animals lacking either a TRPV-related channel, encoded by the gene *osm-9*, or the *tax-4/tax-2*-encoded cGMP-gated channel [36]. *osm-9* mediates attraction to volatile compounds, as well as avoidance and nociceptive behaviors [37], [38]; *tax-4/tax-2* function downstream of the signal transduction pathways for thermotaxis, chemotaxis to soluble stimuli, aerotaxis, and chemotaxis to a different set of volatile compounds than those sensed by *osm-9*-expressing neurons [36]. As shown in Table 1, we failed to detect a significant divergence from wild-type behavior in *osm-9* mutants (*p* against the CRW model  =  0.0008, Kolmogorov-Smirnov test; Figure S3C).

By contrast, mutations in *tax-4* and *tax-2* resulted in a complete loss of directionality (*p* against the wild type  =  0.008, Kolmogorov-Smirnov test; Table 1, Figure S3D-F). We therefore asked whether specific signal transduction pathways upstream of *tax-4/tax-2* could recapitulate the phenotype of the channel mutation. First, we assayed *gcy-8*; *gcy-18*; *gcy-23* triple mutants, defective in three guanylyl cyclases essential for thermotaxis [39]. As shown in Table 1, these animals exhibited wild-type directional behavior (*p* against the CRW model  =  0.0003, Kolmogorov-Smirnov test; Figure S4A). An aerotaxis mutant, *gcy-31; gcy-33; gcy-35*, also displayed no observable defect (*p* against the CRW model  =  0.003, Kolmogorov-Smirnov test; Figure S4B). Furthermore, genetically ablating the main cellular mediators of aerotaxis did not result in loss of directionality (*p* against the CRW model  =  0.0002, Kolmogorov-Smirnov test; Table 1, Figure S4C).

Targeting chemotaxis towards soluble as well as volatile compounds in *tax-4/tax-2*-expressing neurons also resulted in no observable defect. Mutants with lesions in *odr-1*, a guanylyl cyclase required for odorant sensation [40] and phototransduction [41] in *tax-4/tax-2* neurons, exhibited no observable defect (*p* against the CRW model  =  0.004, Kolmogorov-Smirnov test; Table 1, Figure S4D). Likewise, animals mutant for *che-1*, which show defects in chemotaxis towards some soluble compounds [42], [43], displayed wild-type directionality (*p* against the CRW model  =  0.02, Kolmogorov-Smirnov test; Table 1, Figure S4E). In addition, a mutation in *daf-22*, a gene in the pathway regulating synthesis of some *C. elegans* pheromones [44], did not significantly impair directional behavior (*p* against the CRW model  =  0.01, Kolmogorov-Smirnov test; Table 1, Figure S4F). Together with the lack of directional bias we report in Figures 3 and S2, these results suggest that while sensory neurons seem to play a role in directional locomotion of *C. elegans*, no single tested sensory modality is required for this behavior.

## Discussion

This study presents a characterization of the paths of *C. elegans* under starvation conditions. We have found that individual *C. elegans* are able to maintain a directional course over times of several tens of minutes, in the apparent absence of external stimuli. Such directional behavior is unlikely to derive from local correlations in direction, as it persists over larger times than the typical timescale of orientation memory, and is not accounted for by isotropic, random models of locomotion. Furthermore, our scaling analysis indicates that the motion of *C. elegans* on the scale of a minute is less directional than that exhibited over longer (∼10 minutes) intervals.

The lack of directionality displayed by *tax-4/tax-2* mutants suggests the possibility that sensory neurons are required, at least at some level, for long-range directional persistence. Could a sensory cue, therefore, promote this behavior? Surprisingly, our studies seem to argue against this possibility. As shown in Figures 3 and S2, directionality of locomotion is not correlated with the spatial position of the apparatus, suggesting that neither the camera nor the scanner set-ups emit directional cues biasing locomotory patterns. The data shown in Figure 3 also indicate that directional cues within the assay plates, including a putative radial gradient, are unlikely. These observations are strengthened by our findings that inhibition by mutation of individual sensory modalities does not affect directional movement. Indeed, as shown in Table 1, mutations blocking chemotaxis, odortaxis, thermotaxis, phototaxis and aerotaxis do not impair long-range directional movement.

The details of our experimental design also suggest that if sensory cues were relevant, they would need to operate at the limits of detection by *C. elegans*. In the camera set-up, for example, temperature gradients do not exceed 0.01°C/cm, 100-fold less than gradients typically applied in thermotaxis assays [18]. Volatile odorant gradients in our set-ups, if these exist, are likely to be on the order of picomolar/cm (assuming diffusion coefficients on the order of 0.1 cm^2^/s, which is a typical range for the small compounds known to attract *C. elegans* [45]. Such shallow gradients are at the limit of detection for *C. elegans* [46].

Water soluble chemicals are expected to diffuse 10^4^ times more slowly than air-borne chemicals of equivalent size, and may thus create more prominent gradients in our assays. Such gradients, however, would require days to form. *C. elegans* has also been reported to track slowly-rotating and static electric fields. The measured electric potential in the camera set-ups, however, is on the order of 10 mV, which is three orders of magnitude smaller than is required to elicit electrotactic responses [31]. In addition, the electric potentials in the camera and scanner set-ups oscillate, respectively, at frequencies of 60Hz and ∼50 kHz, well beyond the tracking capability of the animal [31].

Another possibility is that *C. elegans* locomotion is guided by self-secreted cues. The speed at which *C. elegans* migrates on the plate suggests that such cues would likely be volatile. However, mutations in the *osm-9* and *odr-1* genes, which block sensation of many volatile compounds, do not affect long-term trajectory heading. Inactivation of *daf-22*, involved in production of some *C. elegans* pheromones also has no effect. Furthermore, studies of animals placed on plates consecutively fail to detect obvious correlations or anti-correlations in locomotory patterns (Figure 3).

Taken together, our results hint at the possibility that long-range directional locomotion of *C. elegans* might not be driven by sensory stimuli. How could the animal’s nervous system support such behavior? One possibility is that animals assemble an intrinsic sense of direction out of proprioceptive information. Indeed, recent evidence indicates that *C. elegans* possesses at least one neuron that is sensitive to body stretch [47]. Body curvature might convey information on the radius of curvature of the path, which, in turn, could translate into heading information, if animals were also monitoring time. However, such a mechanism cannot account for directional persistence across sharp, discrete perturbations of body shape, such as reversals and omega bends. In this latter case, we would have to posit that the orientation discontinuity is stored at the neuronal level, and that such information feeds back into the circuit controlling locomotion. Remarkably, this is indeed the case for the omega bend, whose features (its amplitude, and its bias towards the ventral or the dorsal direction) appear to be encoded by head motor neurons [26]. Head motor neurons also synapse backward onto interneurons, and might, therefore, act as nodes where control of locomotion is coupled to, and determined by, past locomotory output. In this picture, TAX2/TAX-4 sensory neurons would work not as transducers of external stimuli, but, rather, as a driving force in the circuit, activating the interneuron layer that controls the execution of turns by means of spontaneous patterns of activity. Step-counting mechanisms have previously been described in ants [48], providing evidence that invertebrates are capable of navigation by proprioceptive mechanisms. Nonetheless, elucidating the relevant neuronal circuitry for directed locomotion in *C. elegans* is likely to be a prerequisite for addressing this model in detail.

## Materials and Methods

### *C. elegans* strains and culture methods

Animals were grown on standard nematode growth medium (NGM) plates seeded with the *E. coli* strain OP50 [49]. Unless indicated, animals were cultured at 20°C. We utilized the following N2-derived strains: *che-2(e1033)* CB1033, *osm-9(ky10)* CX10, *daf-19(m86);daf-16(mu86)* CF1108, *tax-4(p678)* PR678, *tax-4(ks28)* OUT87, *tax-2(p691)* PR691, *odr-1(n1936)* CX2065, *che-1(p692)* PR692, *gcy-8(oy44); gcy-18(nj38); gcy-23(nj37)* IK597, *gcy-31(ok296); gcy-33(232); gcy-35(769)* CX6803, *gcy-36*::EGL-1; *gcy-35*::GFP CX7102, *daf-22(m130)* DR476. Strains CX2065, CX6803 and CX7102 were a gift from Cori Bargmann. Some strains were provided by the Caenorhabditis Genetics Center, which is funded by NIH Office of Research Infrastructure Programs (P40 OD010440).

### Behavioral assay protocol

To minimize growth of contaminants, all assays were performed on plates containing a modified NGM medium without peptone. Assay plates (240 mm×240 mm polystyrene culture plates; Nunc, Corning) were poured approximately 1 to 2 weeks before an experiment, and subsequently kept at 4°C in air-tight boxes. The scanner-array wild-type assays (N  =  250 trajectories) were performed on plates poured ∼24 hours before the assays. In one instance, animals were assayed on a batch of ∼3-week-old plates. Animals in these radial preference assays displayed a bias towards the edges of the plates; however, no radial bias was observed when assaying animals on plates poured up to 12 days before the assays (Figure 3). After a set of assays, the agar within the plates was discarded, and the plates were soaked in a sodium hypochlorite solution, rinsed, dried, and reused.

Assays were carried out on 1-day-old adults by selecting L4 larvae on the day before the experiment. To exclude possible animal-to-animal interactions, a single individual was assayed per plate. Before each assay, bacteria were removed from an animal’s body by transferring it twice in succession to individual unseeded NGM plates. Animals were then left to crawl on an unseeded agar plate for about 10 minutes, before being transferred to the center of the assay plate. In radial preference assays, animals were started from a point about 4.5 cm away from the plate center. Assay plates were sealed with Parafilm (Cole-Parmer) just before commencing image acquisition.

### Imaging set-ups

Images were collected with one of two imaging set-ups: a fixed-camera setup or a scanner-array set-up. On both, images were acquired for 80 minutes. Image sequences were tracked until the animal reached the edge of the plate. On the camera set-up, animals were illuminated in trans with a 24”x 24” edge-lit, diffuse LED light source (“LitePad”, Rosco). Images were acquired with a consumer Digital Rebel 300D Canon camera custom-mounted with a 50mm focal length lens (Beseler, 50 mm f/3.5 Beslar Enlarging Lens) and a neutral density filter (Hoya, NDX4). The camera sensor (6.3 megapixels) was placed at a working distance of 3 ft. from the glass stage holding the plates, resulting in a resolution of ∼100 pixels/mm^2^. To enhance contrast, we further equipped the stage with two light control films (Vikuiti, 3M), placed at right angles. The camera was controlled by custom time-lapse software developed with Microsoft Visual C++ using Canon SDK (EDSDK 2.5). Images were acquired at a frame rate of 1 image/1.5 seconds. All experiments were performed in a temperature- and humidity-controlled room (T = 22.0 +/− 0.2°C, humidity: 54% +/− 2%). The typical temperature difference at different points on the stage in contact with the edges of the plate was estimated by measurements with a thermistor (“Stainless Steel Temperature Probe”, Vernier) to be within 0.1°C. Images collected with this set-up were segmented using a custom script in Image-Pro Plus (Media Cybernetics), and successively tracked with custom MATLAB code (MathWorks) adapted from the Parallel Worm Tracker [50].

The scanner-array set-up comprises 10 flatbed CCD scanners (EPSON Perfection 2400 Photo) connected to a single PC running Linux (Ubuntu 8.04) and interfaced using the SANE application programming interface (http://www.sane-project.org/). Scanners are directed via a PERL script to acquire images at a resolution of 300 dpi, corresponding to about 0.4 pixels/cm, at a frame rate of 1 image/20 seconds. Heat dissipation from the internal cold-cathode fluorescent lamp causes the scanner surface to heat to temperatures that are noxious to *C. elegans*. We therefore modified the scanners to allow for fan-driven airflow across the scanner body, thus reducing the temperature difference across opposite sides of each scanner, relative to the fan, to ∼1°C. Assay plates were positioned on the scanner body face-up, and covered with black felt to improve contrast. Image sequences collected with this setup were segmented in ImageJ (http://rsbweb.nih.gov/ij/) and tracked with the ImageJ MTrack2 plugin developed by N. Stuurman (http://valelab.ucsf.edu/~nico/Ijplugins/Mtrack2.html).

### Trajectory analysis

**Correlated random walk (‘CRW’) model.** Each experimental path was first sub-sampled at its correlation time, defined as the first zero-crossing of the autocorrelation function of the velocities. We then computed the distributions of step sizes and turning angles of the resulting path, and constructed synthetic trajectories by drawing at random from those distributions for an equal number of steps as the sub-sampled path. The synthetic net displacement, squared, is the sum of two Gaussian-distributed variables, and thus follows chi-square statistics with two degrees of freedom, providing an immediate validation for our algorithm [51]. Sets of samples of the scanner-array data-set of directionality indices were drawn from the data with replacement.

**‘Mirror’ model.** To build ‘mirror’ synthetic trajectories, we segment trajectories into ‘runs’ and ‘turns’. We label as ‘turns’ instances with angles between consecutive instantaneous directions whose cosine is less than a threshold value of 0.75. We then cluster together turns spaced less than 6 seconds apart from each other, and label them as a single turning event. Sets of mirror trajectories are then obtained by reversing the sign of the angles corresponding to a turning event, with probability ½.

**‘Coarse-grained’ trajectories.** Coarse-graining lengths were computed by sampling the paths at intervals of size *δ*. The first sampled point was varied between the first time point and the time point *δ*, and the coarse-grained length at a scale *δ* was defined as the average coarse-grained length over these initial conditions. To compute the local slope of the plot of the logarithm of the coarse-grained length versus the scale *δ*, we first interpolate the function with a cubic spline. We then fit a first-degree polynomial to the smoothed function on overlapping windows of fixed size (in log-space). Finally, we interpolate with a cubic spline the value of the slopes obtained over the different windows. Slopes for the null model are obtained by fitting the plot of the logarithm of the coarse-grained length versus the scale *δ* for individual realizations of the null model. From the resulting distribution, we obtain the slope’s average and its variance. The value of the slope at long times (*δ* ∼ 8 minutes) is computed by evaluating the interpolating spline at *τ  = 400 s*, or else at 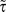 *= T/4,* for those trajectories with a total duration *T* shorter than 4*τ.*

## Supporting Information

Figure S1 **Scanner trajectories display long-range directional persistence.** (**A**) Trajectories of representative wild-type animals on the scanner-array set-up. Red dots on the trajectory indicate the position of the animal at 10-minute intervals. (**B**) Histograms of radial displacements for the trajectories shown in (**A**) (see Figure 2). (**C**) Heading autocorrelation function (see Figure 2).
(TIF)

Figure S2 **Directionality is independent of plate parameters.** (**A**) Histogram of radial displacements for the whole data set (N = 42 trajectories) acquired with the camera set-up. Directions are relative to the set-up. (**B**) Directionality indices *d* of the paths acquired in radial preference assays (see Figure 3). Full black circles: 12-day-old plates. Empty black circles: freshly-poured plates. Red line: average of a set of 20 samples from the wild type scanner-array data-set (‘scanner average’). Red shading: one standard deviation above and below the scanner average. Blue line: average of a set of 20 samples from the CRW model. Blue shading: average of the CRW model +/− one standard deviation. Gray dashed line: *d* = 0.05. 21% *d*<0.05. *p* vs. CRW model  = 0.0002 (Kolmogorov-Smirnov test).
(TIF)

Figure S3 **Behavior of sensory mutants.** Directionality indices (*d*) of sensory mutants. (**A**) *che-2* ciliary mutant. Black dots: mutant data. Red line: average of 20 samples from the wild-type scanner-array data-set. Red shading: one standard deviation above and below the scanner-array set-up average. Blue line: average of 20 samples from the CRW model. Blue shading: average of the CRW model +/− one standard deviation. Gray dashed line: *d* = 0.05. 7% *d*<0.05. *p* vs. wild-type  =  0.003 (Kolmogorov-Smirnov test). (**B**) *daf-19* ciliary mutant. 12% *d*<0.05. *p* vs. wild-type  =  0.03 (Kolmogorov-Smirnov test). (**C**) *osm-9* channel mutant. 25% *d*<0.05. *p* vs. CRW model  =  0.0008 (Kolmogorov-Smirnov test). (**D**) *tax-4(p678)* channel mutant. 11% *d*<0.05. *p* vs. wild type  =  0.008 (Kolmogorov-Smirnov test). (**E**) *tax-2* mutant. Black dots: mutant data. Red line: average of samples from the wild-type scanner-array data-set (‘scanner average’). Red shading: one standard deviation above and below the scanner average. Blue line: average of 20 samples from the CRW model. Blue shading: average of the CRW model +/− one standard deviation. Gray dashed line: *d* = 0.05. 0% p<0.05. *p* vs. wild-type  =  0.009 (Kolmogorov-Smirnov test). (**F**) *tax-4(ks28)* mutant. 8% *d*<0.05. *p* vs. wild-type  =  0.006 (Kolmogorov-Smirnov test).
(TIF)

Figure S4 **Behavior of signal transduction and pheromone mutants.** Directionality indices (*d*) of sensory mutants. (**A**) *gcy-8*; *gcy-18*; *gcy-23* thermotaxis triple mutant. Black dots: mutant data. Red line: average of samples from the wild-type scanner-array data-set. Red shading: one standard deviation above and below the scanner-array set-up average. Blue line: average of 20 samples from the CRW model. Blue shading: average of the CRW model +/− one standard deviation. Gray dashed line: *d* = 0.05. 25% *d*<0.05. *p* vs. CRW model  =  0.0003 (Kolmogorov-Smirnov test). (**B**) *gcy-31*; *gcy-33*; *gcy-35* aerotaxis mutant. 30% *d*<0.05. *p* vs. CRW model  =  0.003 (Kolmogorov-Smirnov test). (**C**) *gcy-36*::EGL-1 aerotaxis mutant (genetic ablation of oxygen-sensing neurons). 35% *d*<0.05. *p* vs. CRW model  =  0.0002 (Kolmogorov-Smirnov test). (**D**) *odr-1* odor-taxis mutant. 45% *d*<0.05. *p* vs. CRW model  =  0.004 (Kolmogorov-Smirnov test). (**E**) *che-1* chemotaxis mutant. 30% *d*<0.05. *p* vs. CRW model  =  0.02 (Kolmogorov-Smirnov test). (**F**) *daf-22* pheromone synthesis mutant. 40% *d*<0.05. *p* vs. CRW model  =  0.01 (Kolmogorov-Smirnov test).
(TIF)
