# Supplementary figures and images for: Directional Locomotion of C. elegans in the Absence of External Stimuli
Source: PLoS One. 2013 Nov 5;8(11):e78535. doi: 10.1371/journal.pone.0078535 (PMC3818405; doi:10.1371/journal.pone.0078535)

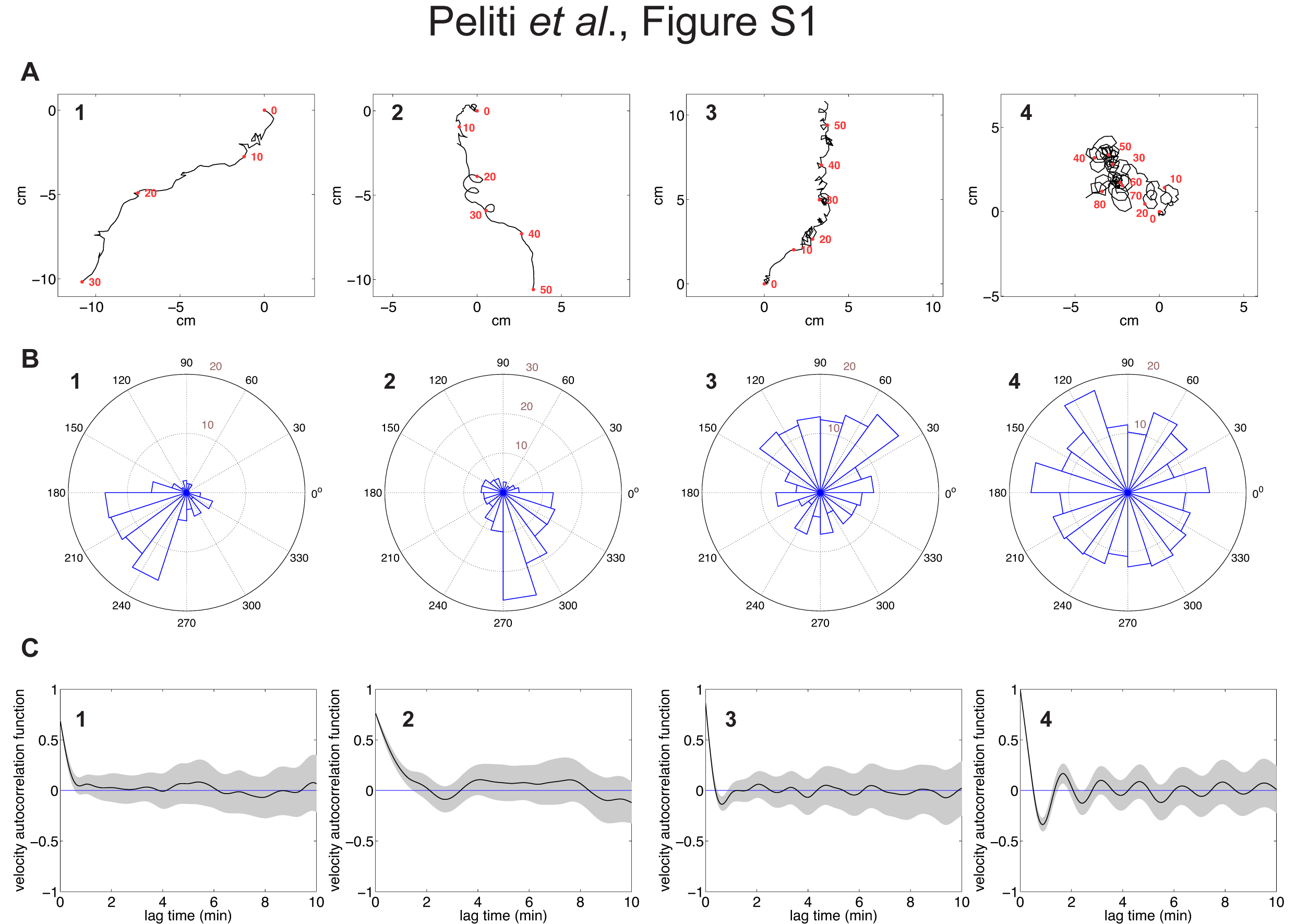

Supplement: Figure S1 — Scanner trajectories display long-range directional persistence. (A) Trajectories of representative wild-type animals on the scanner-array set-up. Red dots on the trajectory indicate the position of the animal at 10-minute intervals. (B) Histograms of radial displacements for the trajectories shown in (A) (see Figure 2). (C) Heading autocorrelation function (see Figure 2). (TIF) [file pone.0078535.s001.tif]

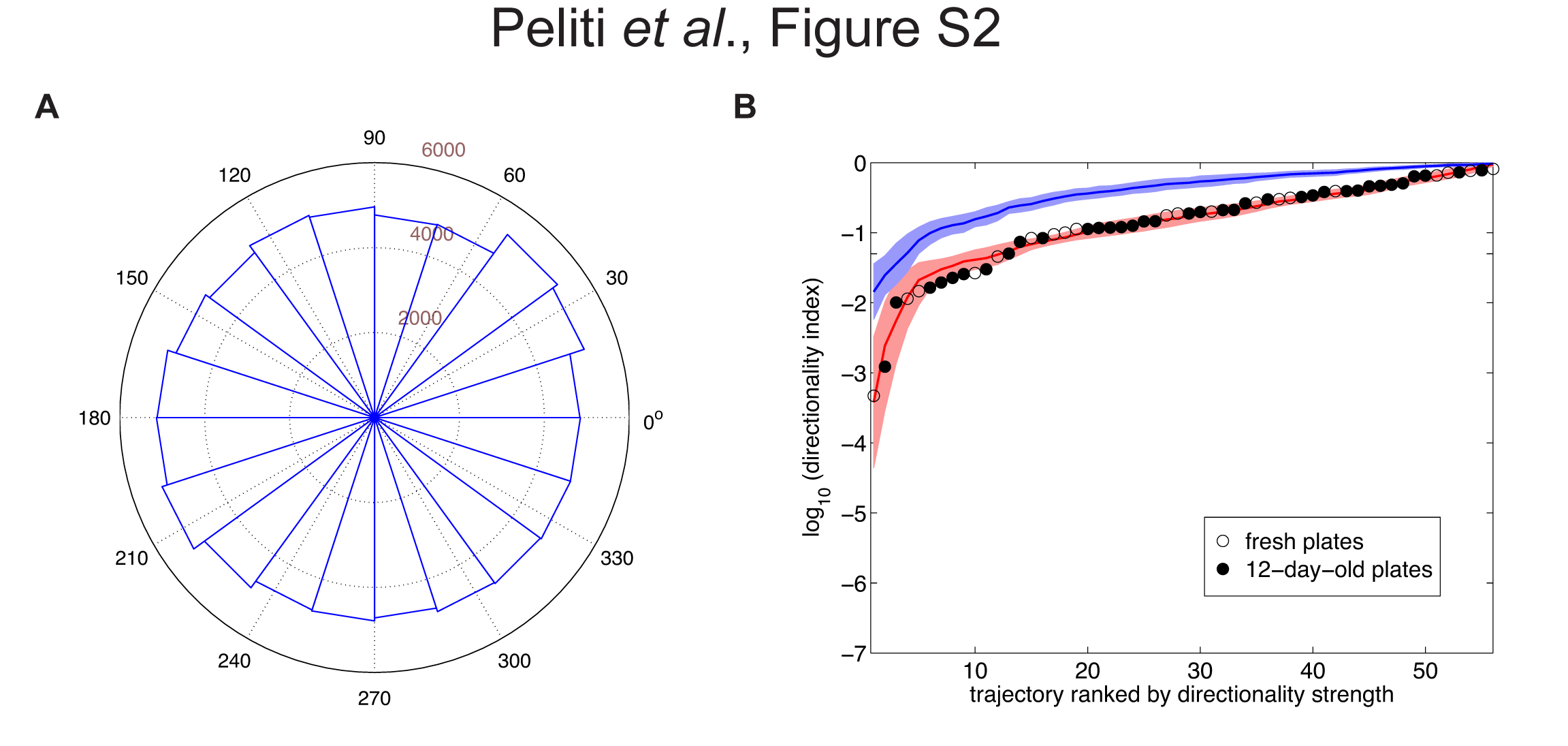

Supplement: Figure S2 — Directionality is independent of plate parameters. (A) Histogram of radial displacements for the whole data set (N = 42 trajectories) acquired with the camera set-up. Directions are relative to the set-up. (B) Directionality indices d of the paths acquired in radial preference assays (see Figure 3). Full black circles: 12-day-old plates. Empty black circles: freshly-poured plates. Red line: average of a set of 20 samples from the wild type scanner-array data-set (‘scanner average’). Red shading: one standard deviation above and below the scanner average. Blue line: average of a set of 20 samples from the CRW model. Blue shading: average of the CRW model +/− one standard deviation. Gray dashed line: d = 0.05. 21% d<0.05. p vs. CRW model = 0.0002 (Kolmogorov-Smirnov test). (TIF) [file pone.0078535.s002.tif]

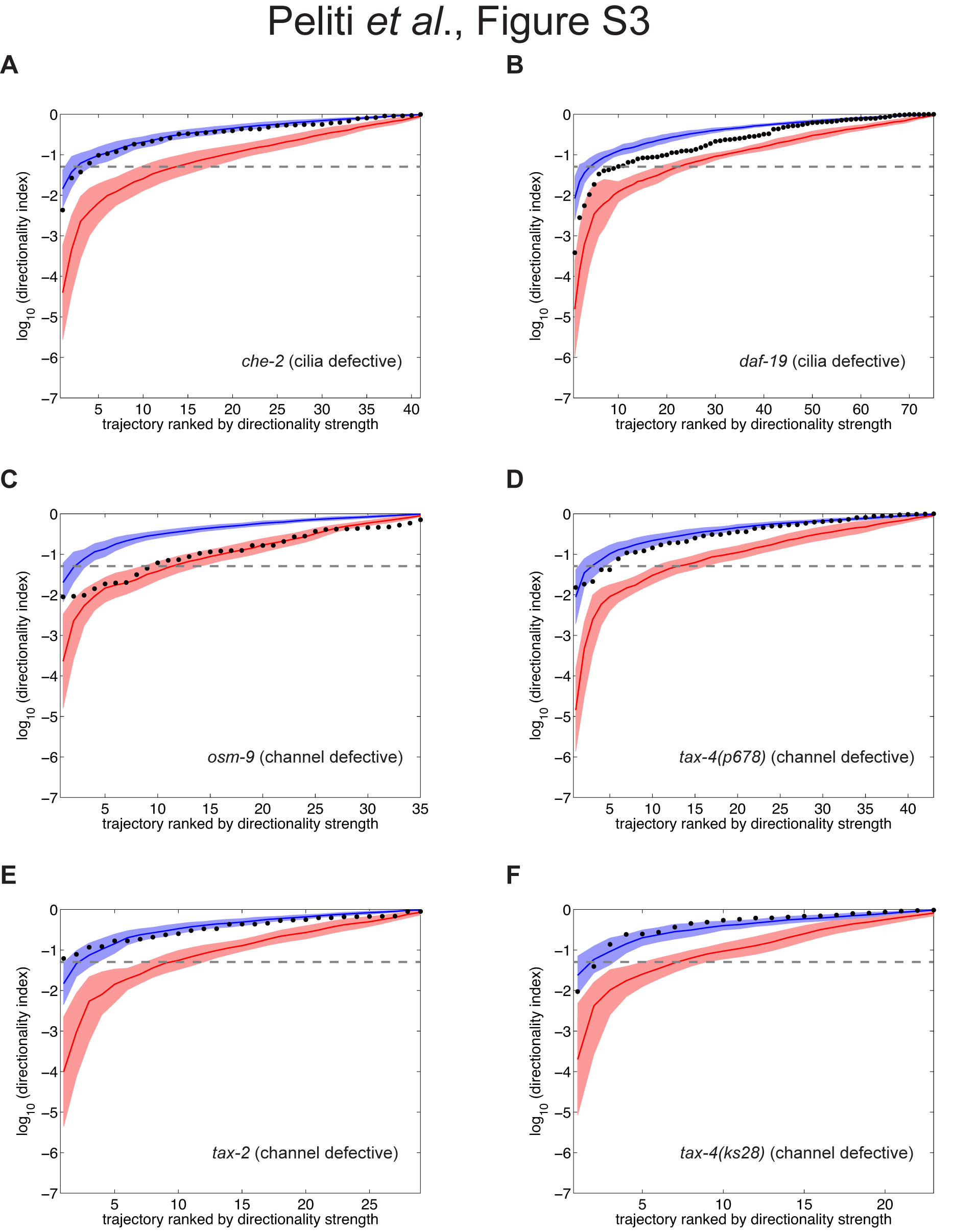

Supplement: Figure S3 — Behavior of sensory mutants. Directionality indices (d) of sensory mutants. (A) che-2 ciliary mutant. Black dots: mutant data. Red line: average of 20 samples from the wild-type scanner-array data-set. Red shading: one standard deviation above and below the scanner-array set-up average. Blue line: average of 20 samples from the CRW model. Blue shading: average of the CRW model +/− one standard deviation. Gray dashed line: d = 0.05. 7% d<0.05. p vs. wild-type = 0.003 (Kolmogorov-Smirnov test). (B) daf-19 ciliary mutant. 12% d<0.05. p vs. wild-type = 0.03 (Kolmogorov-Smirnov test). (C) osm-9 channel mutant. 25% d<0.05. p vs. CRW model = 0.0008 (Kolmogorov-Smirnov test). (D) tax-4(p678) channel mutant. 11% d<0.05. p vs. wild type = 0.008 (Kolmogorov-Smirnov test). (E) tax-2 mutant. Black dots: mutant data. Red line: average of samples from the wild-type scanner-array data-set (‘scanner average’). Red shading: one standard deviation above and below the scanner average. Blue line: average of 20 samples from the CRW model. Blue shading: average of the CRW model +/− one standard deviation. Gray dashed line: d = 0.05. 0% p<0.05. p vs. wild-type = 0.009 (Kolmogorov-Smirnov test). (F) tax-4(ks28) mutant. 8% d<0.05. p vs. wild-type = 0.006 (Kolmogorov-Smirnov test). (TIF) [file pone.0078535.s003.tif]

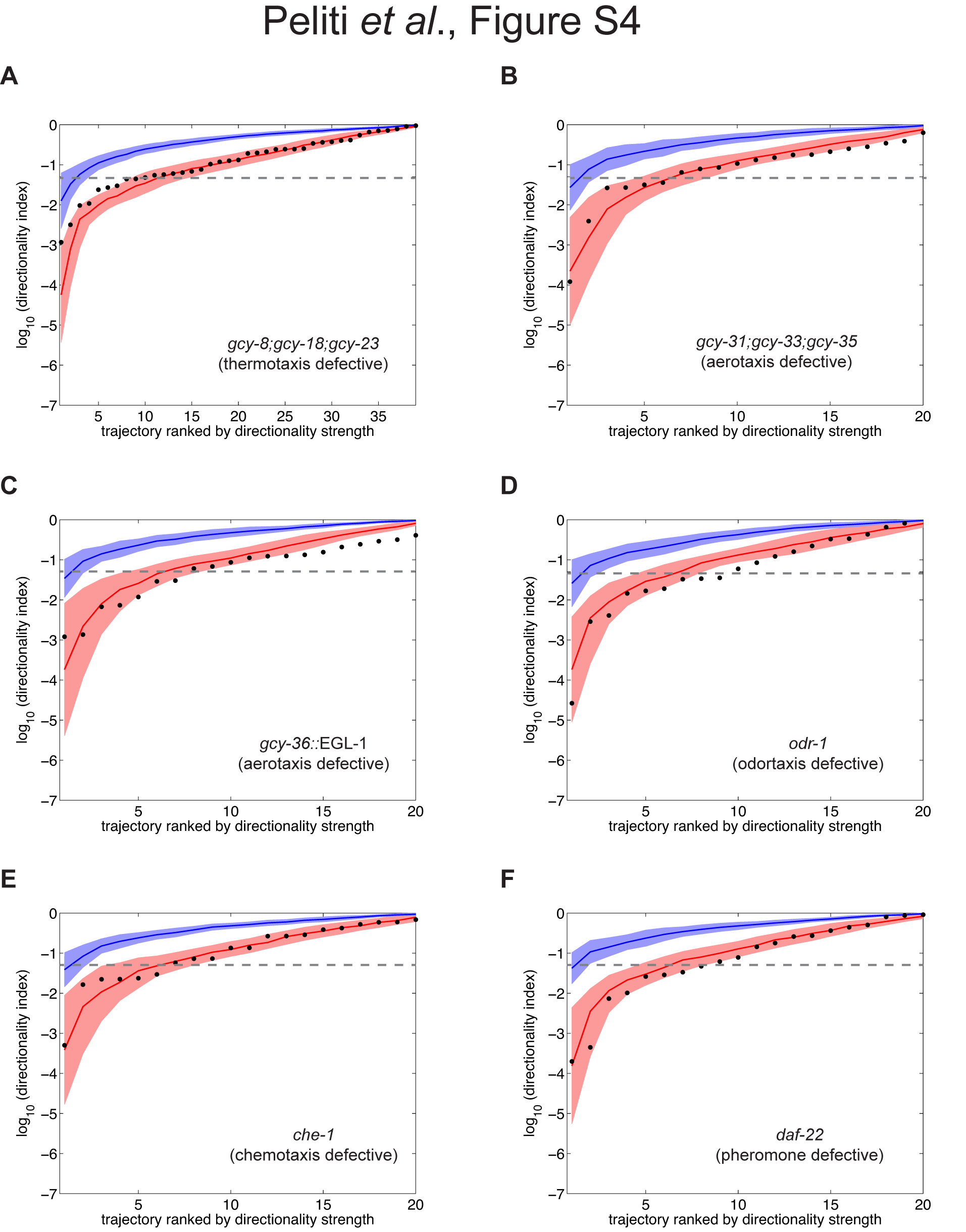

Supplement: Figure S4 — Behavior of signal transduction and pheromone mutants. Directionality indices (d) of sensory mutants. (A) gcy-8; gcy-18; gcy-23 thermotaxis triple mutant. Black dots: mutant data. Red line: average of samples from the wild-type scanner-array data-set. Red shading: one standard deviation above and below the scanner-array set-up average. Blue line: average of 20 samples from the CRW model. Blue shading: average of the CRW model +/− one standard deviation. Gray dashed line: d = 0.05. 25% d<0.05. p vs. CRW model = 0.0003 (Kolmogorov-Smirnov test). (B) gcy-31; gcy-33; gcy-35 aerotaxis mutant. 30% d<0.05. p vs. CRW model = 0.003 (Kolmogorov-Smirnov test). (C) gcy-36::EGL-1 aerotaxis mutant (genetic ablation of oxygen-sensing neurons). 35% d<0.05. p vs. CRW model = 0.0002 (Kolmogorov-Smirnov test). (D) odr-1 odor-taxis mutant. 45% d<0.05. p vs. CRW model = 0.004 (Kolmogorov-Smirnov test). (E) che-1 chemotaxis mutant. 30% d<0.05. p vs. CRW model = 0.02 (Kolmogorov-Smirnov test). (F) daf-22 pheromone synthesis mutant. 40% d<0.05. p vs. CRW model = 0.01 (Kolmogorov-Smirnov test). (TIF) [file pone.0078535.s004.tif]
